# Practices and motives behind antibiotics provision in drug outlets in Tanzania: A qualitative study

**DOI:** 10.1371/journal.pone.0290638

**Published:** 2023-08-31

**Authors:** Pendo M. Ndaki, Joseph R. Mwanga, Martha F. Mushi, Eveline T. Konje, Kathryn Jean Fredricks, Mike Kesby, Alison Sandeman, Stella Mugassa, Msilikale W. Manyiri, Olga Loza, Katherine Keenan, Stanley M. Mwita, Matthew T. G. Holden, Stephen E. Mshana

**Affiliations:** 1 Department of Biostatistics, Epidemiology and Behavioral Sciences, School of Public Health, Catholic University of Health and Allied Sciences, Mwanza, Tanzania; 2 Department of Microbiology and Immunology, Weill Bugando School of Medicine, Catholic University of Health and Allied Sciences, Mwanza, Tanzania; 3 Department of Geography and Sustainable Development, University of St Andrews, St Andrews, United Kingdom; 4 Department of Division of Infection and Global Health, School of Medicine, University of St Andrews, St Andrews, United Kingdom; 5 School of Pharmacy, Catholic University of Health and Allied Sciences, Mwanza, Tanzania; Wroclaw University of Environmental and Life Sciences: Uniwersytet Przyrodniczy we Wroclawiu, POLAND

## Abstract

Dispensing antibiotics without prescription is among the major factors leading to antimicrobial resistance. Dispensing of antibiotics without prescription has negative impact at the individual and societal level leading to poor patient outcomes, and increased risks of resistant bacteria facilitated by inappropriate choice of antibiotics doses/courses. Antimicrobial resistance is a global public health threat which is projected to cause 10 million deaths by 2050 if no significant actions are taken to address this problem This study explored the practices and motives behind dispensing of antibiotics without prescription among community drug outlets in Tanzania. Finding of this study provides more strategies to antibiotics stewardship intervention. In-depth interviews with 28 drug dispensers were conducted for three months consecutively between November 2019 and January 2020 in 12 community pharmacies and 16 Accredited Drug Dispensing Outlets (ADDOs) in the Mwanza, Kilimanjaro and Mbeya regions of Tanzania. Transcripts were coded and analyzed thematically using NVivo12 software. Majority of dispensers admitted to providing antibiotics without prescriptions, selling incomplete courses of antibiotics and not giving detailed instructions to customers on how to use the drugs. These practices were motivated by several factors including customers’ pressure/customers’ demands, business orientation-financial gain of drug dispensers, and low purchasing power of patients/customers. It is important to address the motives behind the unauthorized dispensing antibiotics. On top of the existing regulation and enforcement, we recommend the government to empower customers with education and purchasing power of drugs which can enhance the dispensers adherence to the dispensing regulations. Furthermore, we recommend ethnographic research to inform antibiotic stewardship interventions going beyond awareness raising, education and advocacy campaigns. This will address structural drivers of AMR such as poverty and inadequate government health services, and the disconnect between public messaging and/or policy and the public itself.

## Introduction

Dispensing of antibiotics (ABs) without prescription is one of the major factors contributing to misuse of ABs and increased levels of antimicrobial resistance (AMR) [1]. Irrational use of ABs has negative impact at the individual n societal level, perpetuating poor patient outcomes and increased risks of resistant bacteria, leading to a loss of effectiveness of ABs, and poor patient outcome [2]. Ability to acquire antibiotics (ABs) without prescription has caused excessive consumption of ABs leading to emergence of AMR [3]. Dispensing of ABs without prescription is common across most parts of the world, with very few exceptions such as North America, Northern Europe, and Australia [4]. The situation of dispensing ABs without prescription in low and middle-income countries (LMICs) is particularly alarming. High rates have been reported in Tanzania [5–7], Nigeria [1]), Eritrea [8], Pakistan [9, 10] and Sri Lanka [11] to mention a few.

Antimicrobial resistance is one of the top ten threats to global health [3]. In 2019, AMR was associated with 3.49 million deaths across the world [12]. AMR is projected to be attributable to 10 million deaths by year 2050 if no actions are taken to address the issue [13]. The global rise of AMR has been paralleled by an increase in the antimicrobial consumption mainly obtained from community drug outlets [2]. About 80% of global ABs consumption occurs outside hospital settings [14]. A systematic and meta-analysis review found that 62% of ABs ordered from drug outlets are purchased without prescription [15], with over 50% of these antibiotics (ABs) purchased from drug outlets, often without prescription. In Africa and other low and-middle-income countries (LMICs), community drug outlets often act as the first point of contact for healthcare [16]. In 2015, the World Health Organization (WHO) study of 12 LMICs reported 93% of patients who used ABs with or without a prescription obtained them from drug outlets [17].

Tanzania’s health care system has a pyramidal structure from Dispensary, Health Centres, District Hospitals, Regional Referral hospitals, and Tertiary hospitals. Primary health care (PHC) is available and physically accessible; however, financial hindrances do limit their access, especially for patients who do not have health insurance. The primary health care is readily accessible through health insurance schemes both government and private health facilities. The main insurance scheme covers the employees in both public and private sectors, is called ‘the National Health Insurance Fund’ (NHIF). The vast majority of people who are not employed are covered by another scheme called ‘community health fund (CHF)’ [18]. However, since CHF is still voluntary, most people who are not employed do not have it hence need to use out-of-pocket cash for health services. Nevertheless, the Government is planning to introduce health insurance for all.

Antibiotics are usually being dispensed at the PHC facilities, but occasionally some medicines are out of stock. Therefore, patients are advised to get them from accredited drug dispensing outlets (ADDOs) and community pharmacies as an out-of-pocket expenditure. The Government provides waivers for a small segment of population (e.g. the elderly of 60 years an above, pregnant women, and under-five years children [19].

Previous studies using a simulated patient approach reported dispensing without prescription is widespread in drug outlets in many LMICs, and particularly in sub-Saharan Africa (SSA) including Tanzania, and pharmacies and drug outlets are the main routes to obtain ABs without prescription [6, 7, 20–23].

Despite prohibitive regulations, there is considerable evidence indicating that ABs are commonly dispensed without prescription in many LMICs [23]. In Tanzania, all ABs are prescription-only medicines and can be legally sold in licensed pharmacies and smaller licensed shops known as ADDOs. Pharmacies can dispense all three categories of retailed medicines (narcotics and psychotropic substances, prescription-only medicine, pharmacy only medicines, over the counter (OTC) medicines, and general sales list), while ADDOs are only permitted to retail those drugs on OTC, ADDO prescription, and general sales medicines [24].

Several previous studies on the drivers of dispensing ABs without prescription have focused on the demographic characteristics of the pharmacists including age, gender and education level [25]. A few studies have explored the motives of dispensing of ABs without prescription including one in Nepal [26]. Understanding the drivers behind dispensing ABs without prescription is important in LMICs where drug dispensers in the community pharmacies provide both healthcare services and dispensing of ABs [27].

Furthermore, several studies have described the features of the drug provision landscape that exists in many LMICs [28, 29]. This is in line with findings from our ‘mystery client’ study conducted by our HATUA consortium in three regions in Tanzania (Mwanza, Mbeya and Kilimanjaro), which reported that amoxicillin was dispensed without prescription by 88.2% of drug dispensers across all three regions [22]. Commonly cited reasons for dispensing ABs without medical prescriptions include the ease of access to community pharmacies and drug outlets compared to other health care facilities, expertise and knowledge of dispensers, patients’ trust, misconceptions about ABs and inappropriate AB use among the public, customer pressure, pharmacists’ need to ensure business survival and lack or weak enforcement of regulations [30], lack of knowledge about and poor attitudes towards AB use and provision [21].

Despite the high levels of dispensing ABs without prescription in the community pharmacies and ADDOs in both rural and urban settings in Tanzania, there is limited information on motives behind dispensing ABs without prescription. The previous qualitative study targeted ADDOs only and reported customer demand and habits to be the motives for dispensing ABs without prescription [31]. The primary aim of this study therefore was to explore the motives behind dispensing ABs without prescription in both ADDOs and community pharmacies in rural and urban setting in three Mwanza, Kilimanjaro, and Mbeya regions in Tanzania.

## Materials and methods

This study was conducted as part of the Holistic Approach to Unravel Antibacterial Resistance in East Africa (HATUA) consortium [32]. HATUA aims to address the social and biological drivers of antimicrobial resistance in multiple sites in Kenya, Uganda, and Tanzania. This study draws specifically on the qualitative investigation to explore the dispensing practices and motives behind provision antibiotics without prescription in Tanzania. using in-depth interviews with those working in drug dispensing outlets across Kilimanjaro, Mbeya and Mwanza.

### Study setting

The study was conducted in Mwanza region in Nyamagana, Ilemela, and Sengerema districts; Mbeya region in Mbeya City and Mbeya rural districts; and in Kilimanjaro region in Moshi Municipality and Moshi rural districts, between 2019 and 2020. The regions and districts were selected based on their geographical location in order to cover northeastern, northwestern and southwestern highlands of Tanzania. The purpose of covering these sites was to represent geographic variation of the three sites, and what we can extra-populate about the whole country of Tanzania.

### Research team and reflexivity

An interview guide was developed collectively by international consortium and the project based on previous literature and thereafter used to explore the drivers for dispensing ABs without prescription. Research training was provided to all national research teams by the leading social scientists from the three East African countries and from the United Kingdom. The tool was pretested by interviewing 10 drug dispensers (5 pharmacy dispensers and 5 ADDO dispensers) in Misungwi district, Northern Tanzania. During data collection in Tanzania, a senior social scientist with a doctorate degree and extensive experience in conducting qualitative interviews served as interviewer. The trained social science research assistants (RA) served as a recorder and note-taker. The researchers had no prior relationship with the interviewees. Participants were informed about the nature and purpose of this research by their local district officials in order to foster recognition and trust.

### Study design and sampling

This study adopted a qualitative interpretative methodological orientation. It was based on face-to-face in-depth interviews with drug dispensers. Participants were drawn from a wider sampling frame of drug dispensers in the study sites in order to ensure different types of outlets (ADDOs and community pharmacies). Participants were obtained from the ADDOs and Community pharmacies across the country identified using the global positioning system (GPS) documented previously [7]. Study participants were then sampled using convenience sampling depending on their availability and readiness to where saturation point was reached. Dispensers who were found in their drug outlets during the study visit and those who consented to participate were included in the study. A total of 28 participants from 12 pharmacies and 16 ADDOs were recruited and interviewed. There was no refusal throughout the study. To protect interviewees’ anonymity, we do not disclose any names or other potentially identifying information and instead we indicate more general information, namely the type of drug dispensing outlet they work at.

### Data collection

In-depth interviews (n = 28), lasting between 45 and 60 minutes, were conducted at the premises where the interviewees worked using an interview guide in *Kiswahili*. Interview topics included questions about the services drug dispensers provide to community and their AB dispensing practices (S1 File). The questions asked whether it was common for patients to ask ABs without prescription and incomplete dose. Participants were asked to state their responses to those situations and were finally asked on the motives behind dispensing ABs without prescription.

Conducting interviews at business premises allowed us to contextualize data, as sometimes we had to pause a digital recorder to allow for customers to be served, and we made informal observational notes about these interactions. Near the end of the interviews, participants were invited to report and discuss any topics not yet covered and to ask questions. Interviews were digitally recorded (with permission from the interviewees) and hand-written notes were also taken to complement the recordings; these were written up immediately after each interview and provided a detailed description of the interview situation, including additional information which could not be recorded, such as non-verbal cues and interactions between customers and drug dispensers, which helped to contextualize drug dispensers’ narratives. Raw data are available upon request from the directorate of research and innovation of the Catholic University of Health and Allied Sciences, Tanzania.

### Data management and analysis

Management and analysis of qualitative data was an iterative process. All 28 audio files containing interviews were transferred into the computer, backed-up in another computer in the office, and transcribed verbatim. *Kiswahili* transcripts were translated in English for further analysis. The English transcripts were subsequently checked against the audio by the interviewer for accuracy of the translation. Back translation was done by randomly selecting a few English transcripts and then translating them back to *Kiswahili*. Hand-written notes taken during the interviews were subsequently typed up and included in the interview transcripts.

Content analysis approach that quantifies and analyze the presence, meanings, and relationships of such certain words, themes, or concepts was used for analysis [33]. Transcripts were coded by two investigators to minimize inter-coder variability using NVivo12 qualitative data analysis (QSR International Pty Ltd. Sydney, Australia) software. We used topics in the interview guide to initially categorize and code the data: those topics became the initial codes. The data was systematically and iteratively reviewed by looking into specific information related to topics to ensure an exhaustive set of data support each code. During this process, additional themes that were not captured by the interview guide were identified, and the validity of existing codes was confirmed. Iterative thematic content analysis was then conducted by two independent investigators (in order to cross-check their interpretation of the data). Three major themes emerged from the data were: (i) customers’ pressure, (ii) business orientation of drug dispensers and (iii) the effects of the customers’ economic circumstances on drug dispensing. Contextual information collected during the interviews helped us to interpret the emerging findings by offering accounts of how customers and drug dispensers interact in the course of healthcare seeking. The processing and analysis of qualitative data was systematic, explicit and reproducible, establishing the validation and trustworthiness of the findings [34].

In reporting this study we have followed a checklist for explicit and comprehensive reporting of qualitative studies (in-depth interviews and focus groups) [35]. Quotations throughout this paper are illustrative of experiences reported by the interviewees.

### Ethical approval

The current study obtained ethical approval from the University of St. Andrews, UK (No. MD14548, 10/09/19); National Institute for Medical Research, Tanzania (No. 2831, updated 26/07/19), Mbeya Medical Research and Ethics Committee (No. SZEC-2439/R. A/V.1/30), Kilimanjaro Christian Medical College, Tanzania (No. 2293, updated 14/08/19) and CUHAS/BMC research ethics and review committee (No. CREC/266/2018, updated on 02/2019). Permission to conduct research was obtained from relevant regional and district authorities.

All informants consented to participate in the study after full disclosure through the information sheet on the informed consent document.

## Results

Out of 28 participants, 15 were identified as female and 13 as male. The mean age for male and female participants was 36 years [SD = 14.16 and 12.71 respectively]. Slightly fewer than two-thirds of participants (18 out of 28) had completed form four secondary education and a 5-week ADDO training. Nineteen out of 28 participants in both community pharmacies and ADDOs were employees, with all participants working in pharmacies being employees. The majority of participants (26 out of 28) across both pharmacies and ADDOs had work experience of more than a year (Table 1).

**Table 1:** Drug dispensers’ socio-demographic characteristics.

Characteristics	Male n (%)	Female n (%)	Overall (%)
**Sex**	13 (46.43)	15 (53.57)	28 (100.00)
**Age**			
15–47 (youth age)	9 (32.14)	13 (52.00)	22 (78.57)
48-63(middle age)	3 (10.71)	1 (3.57)	4 (14.29)
>64 (elderly age)	1 (3.57)	1 (3.57)	2 (7.14)
**Education Level**			
ADDO training	2 (7.14)	9 (32.14)	11 (36.67)
Certificate in pharmacy	2 (7.14)	1(3.57)	3 (10.71)
Diploma in pharmacy	6 (21.43)	3 (10.71)	9 (32.14)
Degree in pharmacy	1 (3.57)	0 (0.00)	1 (3.57)
Other training	1 (3.57)	3 (10.71)	4 (14.29)
**Designation**			
Dispenser	9 (32.14)	8 (28.57)	17 (60.71)
Pharmaceutical Assistant	2 (6.67)	1 (3.57)	3 (10.71)
Pharmaceutical Technician	5 (17.86)	2 (7.14)	7 (23.33)
Pharmacist	1 (3.57)	0 (0.00)	1(3.57)
**Outlet level**			
Pharmacy	9 (32.14)	3 (10.71)	12 (42.85)
ADDOs	4 (14.29)	12 (42.85)	16 (57.14)

### Participants’ socio-demographic characteristics

In the course of data analysis, three closely interrelated major themes were identified (Table 2): the pressure from customers on the dispensers when they demand ABs without prescriptions; business orientation which is profit making motivation of drug dispensers; and customers’ economic circumstances. According to drug dispensers, each of these three factors contributed to poor dispensing practices in Tanzania. The high demand for ABs by customers is among the motives behind pressure exerted on dispensers to provide ABs without prescription. Customer pressure is tied to business orientation; dispensers feel that they have to dispense ABs without prescription in order to keep their customers, hence make profit.

**Table 2:** Themes for practices and motives behind dispensing antibiotics without prescription.

Subcategories	Themes	Drug dispensers n = 28 (100)
1.Provided Advice on management of symptoms	1. Customer’s pressure: pressure from customers put on the dispensers when they demand ABs without prescriptions	24 (85.7)
2. Dispensing of Antibiotics		
	2. Business orientation which is profit making motivation of drug dispensers	24 (85.7)
	3. Customers’ economic circumstances.	26 (92.9)
1. Awareness of antibiotic treatment guidelines	Have outdated antibiotics treatment guidelines	10 (35.71)
	2. Do not have access to antibiotic treatment guidelines	15 (53.6)
2. Antibiotic stewardship		
	3. Use of antibiotic treatment guidelines for patients with no prescriptions	3 (10.7)

In the first instance, community pharmacies and ADDOs are business ventures established with the dual aim of providing health service but at the same time making a profit for a living. Our data shows that these two aims can come into conflict and that the customers’ lack of resources further aggravates ‘inappropriate’ dispensing practices. For example, dispensers often claim that they dispense ABs without prescription because they know their customers cannot afford a hospital visit to obtain a prescription.

Also, majority of the visited community drug dispensers were aware of the treatment guideline and the ABs stewardship as indicated in Table 2. However, they continued with the practices because of customers pressure, business orientation and low economic status of their clients.

#### Customer pressure

Majority of drug dispensers reported feeling pressure from their customers to sell ABs without prescription and, during an interview, one dispenser said:

“*If one doesn’t have a prescription*, *it is a challenge*. *Sometimes when I keep on insisting a customer to provide a prescription*, *I get pressurized and end up dispensing the drugs one wants to buy without prescription*.*”* (Pharmaceutical technician 01).

“*Some clients are hard to convince otherwise*. *If you advise them to go for test first*, *they tell you that they know that*, *but it is their money which they want to spend*, *and they insist on buying antibiotics without prescription*. *Consequently*, *I end up selling*.*”* (Dispenser, ADDO 01).

Both community pharmacy workers and ADDO dispensers experience pressure from customers to sell ABs without prescription. One interlocutor remarked:

“*A customer may come here asking for a drug to treat his cough*. *If I tell him that what I have is antibiotics for children above one year old*, *he will not understand and insist that I should give him*. *I do advise him*, *but he would not listen*, *then I have to sell the drug*.” (Dispenser, ADDO 02).

#### Drug dispensers’ business orientation

The second emerging theme was dispensers’ business orientation since dispensers prioritize prof making over ‘healing’ customers. The majority of participants reported selling ABs without a prescription because were ‘doing business’ for maximizing profit. One dispenser described a typical situation as follows:

“*When a customer comes without prescription and needs antibiotics*, *I have to give antibiotics because I am doing business for profit and do not want to lose customers*,*”* (Pharmaceutical technician 04).

To consider profit as mentioned above mean that when you allow clients to buy without prescription, then you will sell more drugs because most clients are likely to come without prescription.

Another informant was even more straightforward in stating the primacy of business considerations; she remarked:

“*This is business*, *we dispense drugs to all*, *those with and without prescriptions*.*”* (Dispenser, ADDO 03).

Furthermore, the majority of participants admitted that dispensing without prescription was closely related to selling less than full course of ABs. Many of them further stated that the main reason was that they did not want to lose customers. Therefore, dispensers agreed to sell incomplete courses of ABs in order to maintain a good business relationship with their customers. One informant said:

“*If I do not sell incomplete courses of antibiotics*, *I will not be in good terms with patients businesswise*, *and they will go to other places and will get the same service which I deny them*. *Therefore*, *I give them so as not to lose business*” (Pharmaceutical technician, 05).

Another informant reiterated the same when she said:

“*If you refuse to give customers what they want and they can afford*, *you will end up destroying the business relationship with them*. *We do give them half course of antibiotics with cautions*.” [Pharmaceutical technician 06].

Another ADDO dispenser reported business orientation as the motive for dispensing ABs without prescription. She said:

“*Other customers want antibiotics for a few days only for example*, *two days*, *and if you tell them that a full course is for five*, *seven or fourteen days*, *they refuse to buy*. *If you keep on insisting them to buy a complete course*, *you lose customers*.” (Dispenser, ADDO 04).

A few dispensers that reported refusing to dispense ABs without prescription stated that they did so to comply with government regulations on drugs dispensing. One dispenser in an ADDO remarked:

“*It happens that customers ask for antibiotic without prescription*, *and I refuse to dispense because the government directive is to come with prescription*, *and there are those who pressurize but I will not dispense*.” (Dispenser, ADDO 06).

#### Customers’ economic circumstances

Drug dispensers also reported that customers’ economic circumstances to their dispensing practices, especially the poverty that many experience. For example, dispensers reported that they often dispensed only a few days’ worth of ABs rather than a full course due to the fact that many customers do not have enough money to afford a complete course. One seller noted:

“*There is half and full course*. *If a customer doesn’t have enough cash*, *I will give him at least half course but not a day’s dose for instance for Ampiclox dosage which is 15 pills for 5 days*.” (Pharmaceutical technician, 09).

The majority’ of ADDO dispensers/community pharmacy workers said they dispensed ABs without prescription or half course because of poverty or low socio-economic status of their customers. One informant remarked:

“*It happens that I sell half course to customers due to their low socio-economic status*, *and advise them to come to buy the remaining half in order to avoid drug resistance*” (Dispenser, ADDO 08).

In the same vein, other informants said:

“*Yes*, *I do sell half course of antibiotics due to the economic constraints of our customers*. *Some don’t have enough money for a complete course*, *and some have already started medication and they want the remaining half to complete the course*. *If I sell half course*, *some do come to take and finish the remaining half and some don’t*.” (Dispenser, ADDO 10).

### Awareness of antimicrobial resistance, antibiotic treatment guidelines and stewardship

All participants were aware of the antimicrobial resistance, treatment guidelines and antimicrobial stewardship. However, the majority of the participants could not access Standard Treatment Guidelines and Policy Guidelines for Implementing Antimicrobial Stewardship.

“*Though I am aware of the antimicrobial resistance*, *treatment guidelines and stewardship… but I do not access the treatment guidelines whenever needed”*. (Dispenser, ADDO 02).

About one third of the participants reported to access the outdated guidelines which discourages them to make as reference when dispensing antibiotics.

“*Though I am aware and know the importance of treatment guideline*, *but I do not use the available versions because I get them while already outdated”* (Pharmaceutical technician, 09)

Few participants less than quota reported to use the updated treatment guidelines for patients who do not have prescription.

“*I use the treatment guidelines for patients who do not have prescription so I can decide on the pattern of antibiotics to be dispensed to clients according to their medical symptoms”*. (Pharmaceutical technician 04).

## Discussion

This paper presents our findings from in-depth interviews with drug dispensers in three sites in Tanzania. Three major themes were identified namely customer pressure, dispensers’ business orientation and customers’ economic circumstances. Furthermore, this study reports drug dispensers’ perceptions of how these factors affected their dispensing practices, in particular their propensity to dispense ABs without prescription. In this section we further contextualize the findings in relation to existing research and suggest how they might be used to design more effective regulatory interventions.

The current study found that drug dispensers believe that customer pressure is responsible for pushing many dispensers into selling ABs without prescriptions. Drug dispensers consistently reported experiencing pressure from their customers to sell ABs without prescription across all study sites and types of outlets. More importantly, this conception of customers’ pressure which we found to be one of the motives behind selling ABs without prescription is similar to what was found elsewhere [23, 36, 37]. However, strong pressure from pharmacy owners on dispensers is a stimulus that favored inappropriate dispensing so as to maximize profit, which was found in other studies [31, 38–40].

The fact that drug dispensers yielded to customers’ demands does not suggest that they lack knowledge or inexperienced or are irrational in their dispensing practices because they did not mention. On the other hand, our study suggests that drug dispensers command high respect in their communities as healthcare providers; dispensers reported that customers frequently visit drug dispensing outlets not only to request specific drugs, but also to describe their symptoms and ask for advice about appropriate treatment.

Drug dispensers in this study also reported dispensing ABs without prescription due to the need to ensure the survival of their business. Although dispensers claim that they are guided by community service, their actions indicate otherwise. However, this presentation of their activities was contradicted both by descriptions of their own practices given by the drug dispensers themselves, and by the objective observational data captured in our mystery client studies reported elsewhere [6, 7]. The business-orientation of drug dispensers is evident in their practices of selling without prescription even incomplete courses of antibiotics. Existing research likewise documented that financial gains motivate drug dispensers to dispense ABs without prescription in Tanzania and other LMICs [21, 31, 39–42]. As pointed out earlier in this paper, we argue that the business-oriented motives of drug dispensers lead to inappropriate dispensing practices. Likewise, drug dispensers in the present study reported that it was often their customers’ economic circumstances–their lack of financial means–that led them to request partial/incomplete courses of ABs.

Other studies have also shown that socioeconomic circumstances of customers are directly related to higher levels of dispensing without prescription [31, 38, 43]. The reason for this may be that for many people the lack of necessary financial resources to consult physician make pharmacies and ADDOs their first and at times only points of contact with the health system [44]. In addition, many drug dispensers admit to being more complacent in dispensing ABs against regulations to customers in this situation.

Our findings have important implications for antimicrobial stewardship (AMS) interventions in Tanzania because when drug dispensers dispense according to regulation, it will reduce antimicrobial resistance and spread of infections; and promote right use of antimicrobials as recommended in the Standard Treatment Guidelines and Policy Guidelines for Implementing Antimicrobial Stewardship [45, 46]. A better understanding of the reasons and motivations behind drug dispensers’ inappropriate dispensing practices, particularly dispensing ABs without prescription will help to come up with strategies to promote optimal dispensing practices.

Despite using convenience sampling the study made efforts to represent different regions, with different socio-demographic and economic backgrounds in Tanzania. The finding of this qualitative study with small sample size of 28 informants is important since it is “illustrative” of the practices of drug dispensers in pharmacies and ADDOs in Tanzania. This snap shot study can help to provide valuable insights to assist in designing future in-depth ethnographic studies [47]. This study demonstrates the value of rapid qualitative methods which could be implemented elsewhere to complement unclear aspects of preceding quantitative study as well as having its own scientific merits.

Our study had some limitations, we had a sample of 28 drug dispensers hence our sample is not generalizable as they are limited to six districts in three regions of Tanzania. The study was a snap-shot assessment of the situation, it could be better if we could have more time to spend with the drug dispensers in longer-term ethnographic work for more in-depth understanding of their perspectives.

## Conclusion

This study used a qualitative approach to explore the drivers of dispensing ABs without prescription. The study finding revealed a range of motivations and motivators for dispensing ABs without prescription among drug dispensers. This included (i) pressure from customers’ demands. Demand for ABs from the side of customers (patients) points out towards the need for public health messages to discourage unnecessary demands for ABs. On the supply side,

(ii) Business orientation of drug dispensers: Free market ABs sale that do not comply with government regulation on ABs dispensing are driven by the need to make profit on drug dispensers’ side. This economic reality is true as selling drug is considered to be a very competitive business and this has bearing on the way drug dispensers operate. The problem of non-regulation ABs sales points out towards the need for joint up intervention that addresses both the supply and demand side of ABs sale.

(iii) Customers’ socio-economic circumstances: This low socio-economic status influenced the ways in which dispensers dispensed ABs. Poverty is considered to be one of the structural drivers of AMR as recently reported by HATUA consortium [48]. Self-medication and treatment non-adherence were driven by perceived inconvenience of the healthcare system, financial constraints and ease of unregulated ABs access. Generally, there is no adherence to good dispensing practices. Dispensers should be reminded to stick to their professional ethics by upholding good dispensing practices as pointed out above.

These findings support calls and in quantities less than the minimum recommended course for appropriate measures to be taken to motivate drug dispensers to adopt good dispensing practices which is not only emphasized by the law as provide by Pharmacy (prescription handling and control) regulations, 2020 but also as part of AMS interventions programs. However, the facts remain that there is insufficient capacity to enforce regulations and also insufficient motivations for voluntary compliance. The current study focused on the perspectives on drug dispensers in regard to dispensing ABs without prescription. We suggest for future research that can explore the drivers of dispensing antibiotics without prescription in perspectives of patients since they are not only the end user of antibiotics but are among the major stakeholders in addressing AMR.

## Supporting information

S1 File Interview guide. (PDF)
