# Supplementary material for: Practices and motives behind antibiotics provision in drug outlets in Tanzania: A qualitative study
Source: PLoS One. 2023 Aug 31;18(8):e0290638. doi: 10.1371/journal.pone.0290638 (PMC10470936; doi:10.1371/journal.pone.0290638)
Supplement: S1 File — (PDF) [file pone.0290638.s001.pdf]

HATUA Project: Antibiotic Seller In-depth Interview Guide

Note to interviewer:

Address all questions (unless already answered in an early response) and use the probes. Give the respondent chance to give their own, *unprompted* answer to the open question *BEFORE* using the probes. It is important to see *whether* respondents *independently* identify issues that are of interest to HATUA or do so *only after* they are *prompted/probed* to do so.

Demographic data

Completed by researcher		
Type of seller – (TANZANIA and UGANDA)	***Pharmacy within Government facility***	
	Larger hospital	
	Smaller clinic	
	Pharmacy	
	Whole sale	
	Retail	
	Mixed whole sale and retail	
	Duka la Dawa / Drug shop	
	Bigger	
	Smaller	
	Mobile van	
	Street hawker / no premises	
Type of seller – (KENYA)	***Pharmacy within Government facility***	
	Larger hospital	
	Smaller clinic	
	Retail distributers	
	Whole seller	
	Chemist	
	Just chemist	
	Chemist and laboratory services	
	Mobile van	

	Street hawker / no premises							
Name of Location (community/suburb/town)								
Time of interview								
Name of business								
Ask the respondent – record								
Gender								
Age								
Ethnicity								
County / district of origin								
Worker or the owner								
Highest level of education								
Does the respondent have any formal qualification as a pharmacist	Yes		No		What qual.			
Does anyone else associated with the store have a formal qualification as a pharmacist	Yes		No		What qual.		Who is qual.	
Years of experience in drug sales business								

a) Introduction

"I would like to ask you a few questions about the service you provide to your customers and in particularly – about the sale of antibiotics. I am not testing you for 'correct' answers – I am interested in *your* own opinions and everyday experiences – I am interested in what is important to *you* - and I want to understand how the drug sale market works from *your* perspective and in *your* experience.

b) The Service you provide to the community

Pharmacists and drug sellers provide an important service to the community. Before asking you about dispensing practices, I'd like to hear more other ways you help your customers.

1. **Is it common or uncommon for customers to describe a set of symptoms and then ask your advice about treatment?**

    (1.1) Probe – please describe a typical situation

2. **Do you feel confident to offer advice to customers on how to treat their symptoms?**

    (2.1) *Probe* – Why do you feel confident/unconfident?

A key element of your job is to dispense drugs to customers. I would like to ask you more about that.

3. **If customers describe symptoms and ask your advice – do you ever dispense antibiotics as a cure (i.e., antibiotic = drug that fights bacteria)?**

4. **Is it common or uncommon for customers to ask directly for a specific antibiotic – for example Amoxicillin?**

    (4.1) *Probe* – please describe a typical situation

5. **If a customer asks for a specific antibiotic – do you ever ask any questions before dispensing – and if so – what do you ask?**

    *(5.1) Prob*e – Do you ever ask for a prescription?
    (5.2) Probe – Do you ask why they want that particular drug?
    (5.3) Probe – Do you ask about symptoms?
    (5.4) Probe – Do you ask if they are taking anything else?

6. **Is it common or uncommon for a customer to ask for an antibiotic drug without a prescription – and what usually happens in cases when there is no prescription?**

(6.1) *Probe* – please describe a typical situation

7. Is it common or uncommon for a customer to ask for an antibiotic drug and for you to refuse to dispense it?

(7.1) *Probe* – Please describe a typical situation?
(7.2) Probe – Why would you refuse?
(7.3) Probe – Do you ever feel pressured by customers to sell them antibiotics – please explain?

8. Is it common or uncommon for a customer who is buying antibiotics – to ask for only a few days' worth rather than buying a whole course?

(8.1) *Probe* – Please describe a typical situation
(8.2) *Prob*e – Why do you think they ask this?
(8.3) *Probe* – How do you respond to such requests – and why?

c) Governance and AMR

9. Are you aware of any guidelines that affect how antibiotics are dispense or sold?

10. What is your opinion of the current system of regulation of drug sales in this country?

(10.1) *Probe* – Should some types of providers be prevented from selling antibiotics – which ones and why?
(10.2) *Probe* – What new rules would improve the situation - or make it worse?

11. Have you ever experienced a situation where a customer complained that a drug that used to work for a common condition no longer seems to work?

(11.1) *Probe* – Please describe the situation – or give an example
(11.2) *Probe* – What do you think caused the drug to stop working?
(11.3) *Probe* – What was your response to that situation?

12. Have you ever heard of 'antibiotic drug resistance' – and what do you understand by that term?

(12.1) *Probe – Interviewer - Give the respondent time to answer – or explain that they have no knowledge - before giving the definition below*)

Antibiotic drug resistance is where bacteria become immune to antibiotic drugs – and the drugs no longer kill-off the bacteria. It is thought that 'drug resistance' is emerging because antibiotics are being *used too much* and are not being used properly.

13. **Some suggest that anti-biotics should only be obtainable when they are: (i) prescribed by a doctor: (ii) sold by a trained pharmacist and when: (iii) a whole course is consumed (not just a few days' worth). What is your view of these suggestions?**

    (13.1) *Probe* – What would the consequences be for your business?
    (13.2) *Probe* – What would be the consequence for your customers?)

14. **Who do you think is best placed to do something about drug resistance in this country?**

    (14.1) *Probe* – Do you feel that there is anything you can do personally?)

d) The business

To finish, I'd like to ask a few questions about the business.

15. **Which antibiotics are your biggest sellers – and why is that?**

16. **Are there any incentives to sell one kind of antibiotic rather than another – please explain and give examples?**

17. **How often do you restock and where do you source the antibiotics that you sell?**

18. **Do you ever worry that suppliers might sell you fake or out of date drugs?**

    (18.1) *Probe* – can you give me an example)?

19. **There are many places where customers can obtain Antibiotics. Why do you think your customers choose to come to you?**

    (19.1) *Probe* – e.g., is it convenience / price / quality / service / range of drugs?)

20. **Is drug selling a very competitive business – and if so – how do you think that affects the way drug sellers operate?**

We are coming to the end of our interview. Is there anything else you would want to share with us about what we have been discussing or do you have any question? Do you have any questions you would like to ask us – we will do our best to answer them.
Thank you so much for your time and insights.
